# Poloxamer-Based Scaffolds for Tissue Engineering Applications: A Review

**DOI:** 10.3390/gels8060360

**Published:** 2022-06-08

**Authors:** Naiyu Cui, Chun-Yu Dai, Xuran Mao, Xun Lv, Yue Gu, Eui-Seok Lee, Heng-Bo Jiang, Yunhan Sun

**Affiliations:** 1 The CONVERSATIONALIST Club, School of Stomatology, Shandong First Medical University and Shandong Academy of Medical Sciences, Tai’an 271016, China; cuinaiyuuu@outlook.com (N.C.); msdaichuny@outlook.com (C.-Y.D.); mxr021227@outlook.com (X.M.); leslie7552@outlook.com (X.L.); guyue12252022@outlook.com (Y.G.); 2 Department of Oral and Maxillofacial Surgery, Graduate School of Clinical Dentistry, Korea University, Seoul 08308, Korea

**Keywords:** poloxamer, hydrogel scaffolds, biomaterials, tissue engineering, 3D printing

## Abstract

Poloxamer is a triblock copolymer with amphiphilicity and reversible thermal responsiveness and has wide application prospects in biomedical applications owing to its multifunctional properties. Poloxamer hydrogels play a crucial role in the field of tissue engineering and have been regarded as injectable scaffolds for loading cells or growth factors (GFs) in the last few years. Hydrogel micelles can maintain the integrity and stability of cells and GFs and form an appropriate vascular network at the application site, thus creating an appropriate microenvironment for cell growth, nerve growth, or bone integration. The injectability and low toxicity of poloxamer hydrogels make them a noninvasive method. In addition, they can also be good candidates for bio-inks, the raw material for three-dimensional (3D) printing. However, the potential of poloxamer hydrogels has not been fully explored owing to the complex biological challenges. In this review, the latest progress and cutting-edge research of poloxamer-based scaffolds in different fields of application such as the bone, vascular, cartilage, skin, nervous system, and organs in tissue engineering and 3D printing are reviewed, and the important roles of poloxamers in tissue engineering scaffolds are discussed in depth.

## 1. Introduction

“Tissue engineering” aims to create substitutes with special functions for repairing and reconstructing damaged tissues or organs [1]. One of the main approaches is to culture bioactive scaffolds loaded with a specific cell or GF type to mimic the extracellular matrix (ECM) [2], a promising strategy that blends amplified cells into bioactive scaffolds for culture followed by implanting them in vivo [3]. An ideal scaffold should grow a suitable vasculature at the application site to regulate the transport and diffusion of nutrients, oxygen, and metabolic wastes and be able to ignore immune and inflammatory responses [4,5,6]. Therefore, scaffold materials applied in tissue engineering should achieve controllable biodegradability, appropriate mechanical properties, and superior biocompatibility, which are suitable for cell growth, proliferation, and differentiation [7].

Biomaterials are widely applied and play a crucial role in the biomedical field. Recent advances in tissue engineering have also highlighted the importance of biomaterials [8]. Hydrogels are cross-linked 3D-network-structured polymeric materials, which are similar to the ECM, with high water content, elasticity, flexibility, and an easily diffusible biomolecule structure. Hydrogels have become promising biomaterials owing to the unique characteristics of the sol–gel transition, whereby they show the transformation from sol to gel under specific biological stimuli [9]. Poloxamer, or Pluronic, is widely applied in biomedical engineering owing to its superior multifunctional properties [10]. It is a water-soluble non-ionic triblock copolymer [11].

The amphiphilicity, reversible thermal responsiveness, and micellar self-assembly behavior of poloxamers allow the formation of micelles with ordered structures, thereby encapsulating the drug for delivery [12,13,14,15]. In recent years, besides being carriers for drug delivery, poloxamer hydrogels have shown great application prospects in tissue engineering as injectable scaffolds loaded with cells and GFs [11]. For example, bone morphogenetic protein-2 (BMP-2) can promote bone formation by osteoblasts and other cells essential for bone reconstruction [16]. However, there are limitations such as instability and easily disrupted integrity during the delivery process. Poloxamer hydrogel micelles effectively address these problems by loading BMP-2, thus limiting its diffusion, enhancing its stability and bioactivity, and maintaining the integrity of cells and GFs [11,17,18]. The high water content of poloxamer and rheological properties contribute to its injectability, and it is a non-toxic polymer, which can be applied as a non-invasive method to lower risks during surgery [19]. When reaching the target sites, in most cases, the hydrogel scaffolds can provide the mechanical properties of the target tissue to create a suitable microenvironment for cell growth, bone integration, ingrowth, or vascular ingrowth [7,20,21,22,23]. In other cases, they can have a chemotactic effect on stem-cell differentiation, act as a physical barrier to protect cells from degradation, mimic the behavior of the tissue to be repaired for tissue repair [24], or receive mechanical stimuli to further promote intracellular signal cascade responses in the nervous system [25].

**Figure 1:**
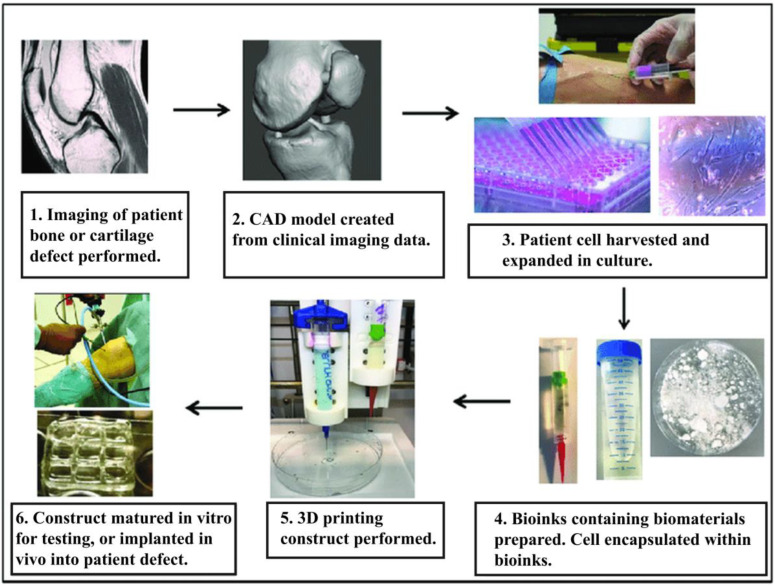
Three-dimensional-printing process. The image has been taken from the publication by Turnbull et al. [28] with permission.

Furthermore, poloxamer hydrogels can be excellent raw materials for 3D printing. Three-dimensional printing is a computer-aided technique that deposits polymer materials in layers to form complex 3D structures during tissue engineering [26], which allows the precise deposition of biomaterials to mimic the desired replacement tissue [27]. The bioprinting process usually begins with the selection of cells and biomaterials for bioprinting structures (Figure 1) [28]. However, the lack of suitable biomaterials for printing and bio-inks is a major hurdle in current development, which requires superior printability to facilitate extrusion and maintain the printed shape. In addition, biocompatibility, low immunoreactivity, and high resolution are required during printing [26]. Poloxamer hydrogels are printable, biocompatible, and inexpensive; thus, they have been successfully applied in 3D printing [29].

However, poloxamer does not fully meet all the criteria of ideal bio-inks, such as low cell survival and viability in vitro [7,30], and hydrogel scaffolds have the limitation of poor mechanical properties. Therefore, appropriate modification of poloxamers to create composite scaffolds or bio-inks with superior properties is the focus of several studies. These modifications include blending poloxamers with various additives and nanocomposites, such as hyaluronic acid (HA), oligopeptides, and alginates, which successfully improved the quality of the hydrogel scaffolds [31,32,33,34].

Owing to these multifunctional properties, poloxamer hydrogel scaffolds have been widely used in bone, vascular, cartilage, skin, nervous system, and organs, such as lung and brain tissue engineering. However, most studies mainly focused on discussing the role of drugs loaded with poloxamer in tissue engineering, with little consideration of the role of the carriers themselves. To the best of our knowledge, no study has specifically addressed the topic of “tissue engineering” and shed light on the effects of poloxamer separately.

In the present review, cutting-edge advances and research on the application of poloxamer-based scaffolds as multifunctional bioactive scaffolds in the emerging tissue engineering field and 3D printing are reviewed, with the aim of gaining insight into the vital roles played by them in tissue engineering and providing a relatively rational reference for biomaterial scientists to adjust the properties of tissue engineering scaffolds and design optimized tools.

## 2. Physical and Chemical Properties

**Figure 2:**
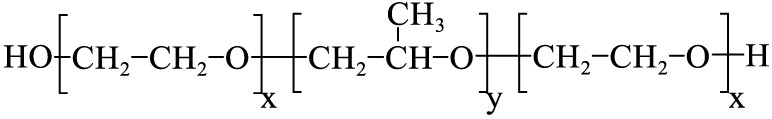
Poloxamer formula: X and Y represent Poly (ethylene oxide) (PEO) and Poly (propylene oxide) (PPO), respectively: Length of PEO and PPO chains. The image has been taken from the publication by Russo et al. [11] with permission.

A poloxamer is a polyethylene oxide-polypropylene oxide-polyethylene oxide triblock (ABA) copolymer [PEO_x_- PPO_y_- PEO_x_] (BASF, Ludwigshafen, Germany) (Figure 2) [35]. The physical state of the poloxamer at room temperature is indicated by the uppercase letter before the number in the name: sheet (F), paste (P), or liquid (L). For example, L61 exists as a liquid and P181 as a paste, where the molecular weight of propylene oxide (PO) can be approximated by multiplying the first or second number by 300, and one-tenth of the weight percent of ethylene oxide (EO) in the copolymer is represented by the last number [36]. Poloxamer has a molecular weight between 1100 and 14,000, which can determine its three physical states at room temperature to a certain extent and can also provide more than 50 amphiphilic, water-soluble, and polycrystalline materials [11]. Because the PO chain is hydrophobic and the EO chain is hydrophilic, different composition ratios (1:9–8:2) have a certain effect on the water solubility of the poloxamer [37]. Because of these differences, poloxamers can be involved in the development of many new drugs and biomaterials and perform different functions in the human body.

**Figure 3:**
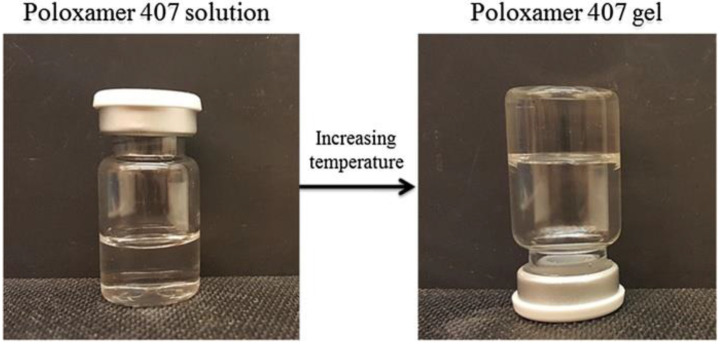
Sol-to-gel transition of poloxamer 407. T_sol–gel_ in thermo-sensitive gels is dependent on the polymer concentration. The image has been taken from the publication by Fakhari et al. [38] with permission.

The polar shell is hydrophilic PEO and its core is hydrophobic PPO, which leads to its special performance in aqueous solution amphiphilicity. It is a nanometer-sized structure that develops at the critical micelle concentration (CMC) as well as the critical micelle temperature (CMT) [11]. Poloxamers can be transformed from a thermally responsive solution to hydrogels, and this process is reversible (Figure 3). Simultaneously, this property can induce a solution to form solid hydrogels. In the study by Fakhari [38], the gelation phenomenon is reversible and characterized by a sol–gel transition temperature (T_sol–gel_). T_sol–gel_ is concentration-dependent and is increased by decreasing the concentration of Poloxamer 407 in an aqueous solution until a lower level is reached, at which point Poloxamer 407 is no longer gel (Figure 3).

Since the 1950s, this feature has been extensively studied in the field of medication development [39,40,41]. At low temperatures, the poloxamer is an unstructured, low-molecular-weight solution. When the temperature and degree of aggregation of the copolymer increase, the poloxamer self-assembles into spherical micelles (micellization), resulting in this thermodynamic characteristic. Because the PEO units expand owing to hydration and remain outside the micelle, while the poly (propylene oxide) units dehydrate inside the created spherical micelle, micellization occurs [42]. The critical micelle concentration (CMC) is another important component of this process. Poloxamer solutions with concentrations higher than CMC form hydrogels at temperatures higher than the solution-hydrogel temperature (critical micelle temperature) [7]. With an increase in the number of (propylene oxide) units and CMT, the CMC value shows a downward trend. Therefore, when the polymer contains more hydrophobic PPO and the concentration and temperature are lower, micellization is more likely to occur. One of the remarkable uses of poloxamers is to cross-link micelles. The key properties of poloxamer cross-linking include physical entanglement, hydrogen bonding, and hydrophobic interactions. Owing to the presence of micelles, the poloxamer macromolecular crosslinking agent has high ductility and toughness. The pH-sensitive drug-release system for cancer therapy consists of bioreducible cross-linked poloxamer micelles (approximately 150 nm in size). Poloxamers have properties other than micellization. The characteristics of typical poloxamer copolymers are listed in Table 1.

**Figure 4:**
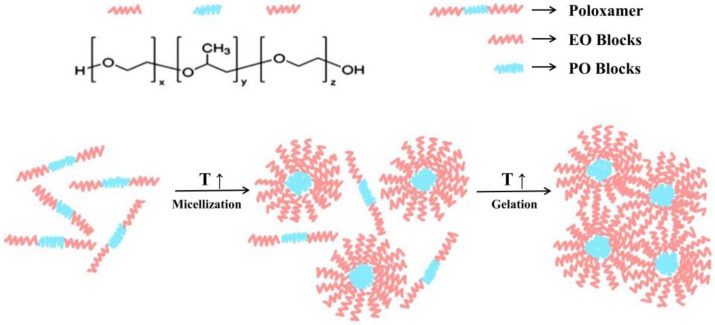
Poloxamer gelation process. The image has been taken from the publication by Zarrintaj et al. [7] with permission.

The flow behavior of aqueous poloxamers varies from Newtonian rheological behavior to viscoelastic rheological behavior and then to unstable rheological behavior depending on temperature and concentration changes [7]. The temperature at which the poloxamer hydrogel shrinks and ruptures is defined as the hydrogel temperature (Th), and it is used as the limit of the temperature change. Individual micelles exist in solutions at low temperatures and medium concentrations. An increase in concentration or temperature results in an increase in the density of the micelles, causing the PEO shells to superimpose and behave as thermoplastic hydrogels (Figure 4) and yield stress. At higher temperatures, the PEO shell shrinks as a result of dehydration, leading to the collapse of the gel structure [43]. The rheological properties of poloxamers have led to their application in injectable stents and bio-inks [44,45]. In the research of poloxamer in situ hydrogels, the injectability and stability of hydrogels are critical elements [14]. Gelation time is the time of the change from a liquid to a hydrogel over injection at a constant temperature (36.5 °C). The completed preparation is transferred into a scheduled syringe, typically with a 20-gauge needle, to hold the volumes securely in place and deliver the in situ hydrogels to the targeted area. Lower than the threshold for gelation, the solutions pass easily to the syringe, whereas higher than the gelation level, the solutions have difficulty passing the syringe needle and dosing is impracticable [46,47,48]. After injection, a rapid transition from liquid to hydrogel can better accommodate the tissue at the site of injury, eliminate free space, and form a template for tissue regeneration. Consequently, the study of the rheological characteristics of poloxamers provides a basis for studying the formulation of its hydrogel preparations.

**Figure 5:**
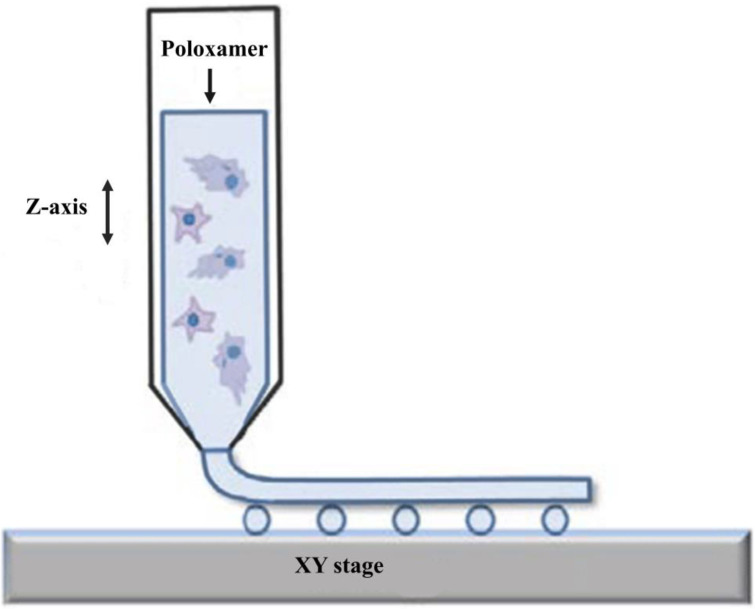
Schematic illustration of the fabrication process for the obtaining of cell-laden poloxamer constructs. The image has been taken from the publication by Gioffredi et al. [27] with permission.

The printer controls the temperature during input and output to ensure changes in the flow behavior of the poloxamer; therefore, the poloxamer acts as a bio-ink to adjust the 3D-printing conditions to obtain a suitable product [49]. The printing equipment consists of a heated dispensing head terminating with a nozzle, an XY stage for the positioning of the dispensing head, and z-axis for controlling its distance from the stage (Figure 5). The cells were well distributed in the scaffolding filaments, and cell viability was maintained throughout the 3D-printing process [27]. The purification/modification process can alter the rheological properties of poloxamers. Tuning rheological properties according to the desired conditions for injectable scaffolds and 3D printing has also become a popular research topic.

Available toxicological evidence suggests that the 50% lethal dose (LD50) of poloxamer, based on oral and skin exposure, is below 5 g·kg^−1^ [50]. It is mainly excreted through the kidneys and secondarily through bile. Poloxamer 181 and poloxamer 407 were assessed as carriers via oral and topical delivery in animals. In high-dose oral tests, poloxamer shows low toxicity but no mortality in animals of different body sizes [43]. The dosage of poloxamer carrier did not show obvious toxicity in animal topical application experiments, and it is considered biocompatible in topical applications. The physicochemical properties of poloxamers include reversible thermal responsiveness [38], providing mucosal adhesion when the ambient temperature changes from room temperature to body temperature, which is widely used for treating dermal and mucous membranes [13]. Because the topical persistence of the formulation lasts longer, this results in an enhanced therapeutic effect. It also provides excellent compatibility with various biological and chemical compounds [51]. As a result, it is employed either as a solubilizer, emulsifier, or stabilizer via the topical route of administration. Poloxamer-based thermosensitive hydrogels have the characteristics of good solubility, good biocompatibility, and no irritation to biofilms [12,52]. The molecular structure, mechanical properties, and bioactivity of poloxamer are customized to simulate the behaviors of different kinds of tissues and are of particular interest in tissue regeneration because of their biocompatibility, low cytotoxicity, and favorable rheological properties [53]. Poloxamer has been used as a matrix for various tissues and organs and has been successfully applied as a scaffold material.

## 3. Poloxamer Scaffolds’ Application in Tissue Engineering

### 3.1. Vascular

The key to the treatment of many vascular and ischemia-related diseases, as well as applications in tissue engineering, is new blood vessel formation. Indeed, angiogenesis and vascular implantation are key to achieving the functional regeneration of complicated tissues through tissue engineering strategies.

#### 3.1.1. Vascular Regeneration

A number of signaling molecules and GFs associated with angiogenesis have been identified; however, owing to their very short half-life in vivo, their clinical application is still very limited. To further enhance the therapeutic activity of these molecules, Ivana et al. proposed a nanocarrier that can integrate the above substances into a solid matrix and release them for a longer period [54].

Poloxamer nanoparticles have excellent pharmacological properties, are stable and nontoxic in simulated biological liquids, and can be effectively freeze-dried for long-term preservation. Furthermore, in vitro cellular experiments have shown that nanosystems can maintain the bioactivity of encapsulated GFs. Under physiological conditions, GFs that degrade within minutes can produce longer-lasting effects, with minimal loss of biological activity. Owing to their small size and favorable injectability, poloxamer nanoparticles do not pose additional dosing problems compared to GF-only solutions.

#### 3.1.2. Wireless Suture

The length of the procedure, technical difficulties, endothelial growth, and foreign-body reactions lead to serious challenges in conventional suturing. Sutureless microvascular anastomosis simplifies microvascular vision, shortens the operative time, and improves surgical outcomes. Tissue adhesive anastomosis is an alternative to the traditional anastomosis. However, several technical challenges are associated with this technology. The sutures were applied precisely to the surfaces of the anastomosis to prevent leakage into the lumen. Poloxamer 407 has been used as a scaffold to solve these problems in previous studies.

A new heparinized poloxamer 407 formulation was developed by Fırat et al. The poloxamer as an endovascular scaffold keeps the lumen open during surgery, promotes sutureless anastomosis of the tissue adhesive to the microvasculature and prevents leakage of the adhesive into the vessel. A therapeutic dose of heparin was added to poloxamer 407 to obtain an antithrombotic intravascular gelatin scaffold. It was applied for sutureless microvascular anastomosis and promises the local release of heparin. For the heparin and poloxamer 407 groups, no heparin-related hemorrhagic complications occurred when poloxamer hydrogel (0.3 mL) containing heparin (150 U) was injected [55]. The addition of heparin may reduce possible early thrombotic complications due to pseudo-endothelial thickening.

Because venous collapse occurs more easily during surgery, the technique required for venous anastomosis is more demanding. Qassemyar et al. also found that poloxamer scaffolds kept the venous cavity open and prevented it from collapsing for difficult-to-anastomotic veins [56]. It also allows for an anastomosis of the vessel stump, avoiding the risk of adhesive leakage into the vascular lumen.

#### 3.1.3. Artificial Vessels

Currently, artificial vessels consist of polycaprolactone (PCL) and polyurethane (PU). PU possesses superior tissue biocompatibility and good elasticity, providing a mechanical system similar to that of natural vascular tissue. Simultaneously, PCL is capable of degrading slowly, exhibiting optimal fracture pressure and providing sufficient suture retention strength for its hydrophobicity and elasticity. PCL and PU are semi-crystalline polymers with biocompatible, non-toxic, and biodegradable properties [57,58,59]. However, one month after the implantation of the beagle model, the artificial blood vessels showed little antithrombotic activity. For the design of vascular grafts with small diameters, it remains a technical challenge to target only the lumen with stable surface treatment for rapid endothelialization and antithrombotic purposes without destroying the artificial vessel.

Early thrombotic occlusion and relatively weak mechanical properties of artificial vessels limited the clinical application. Currently, there are no satisfactory artificial vascular grafts with small diameters that can meet clinical needs.

To improve the electro-spun PU/PCL’s hydrophilicity in a controlled way with minimal impact, A Nguyen-My L et al. blended the non-ionic surfactant poloxamer 407 with PU/PCL [60]. An inner polyurethane/polycaprolactone/poloxamer (PU/PCL/poloxamer) double-layer hollow tube was prepared, which promotes cell proliferation and differentiation while inhibiting the adhesion of platelets for vascular engineering. Double-layered hollow tubes possess good tensile strength and uniform nanofiber distribution. Poloxamers improve the wettability of electro-spun surfaces owing to the hydrophilicity of the polyethylene glycol block. Suitable surface concentrations of poloxamer, ranging from 3 wt.% to 8 wt.%, can provide high bioactive surface structures and bioactive sites to achieve the dual effect of promoting rapid cell growth and inhibiting platelet adhesion/activation initially.

Furthermore, this study raises the possibility of further research into endothelial behavior under dynamic conditions and in contact with blood flow for further development of high-performance vascular grafts.

#### 3.1.4. Temporary Vascular Occlusion

Percutaneous endovascular techniques usually include the restoration of vascular patency; however, it is less common to occlude vascular structures. During this process, commonly referred to as embolization, it may be necessary to temporarily block the normal vasculature to protect the normal vascular bed from embolization agents or cytotoxic agents [61,62].

Poloxamer injections can produce persistent blood-vessel occlusions at different sites. Poloxamer occlusion is mostly transient and dissolves 5–90 min after embolization. Transient occlusion of the poloxamer did not cause significant vascular-wall damage immediately or within one week. These short-term occlusions had no effect on terminal organs. Except for one case of local vena cava obstruction, poloxamer embolization did not affect the clotting time, and no thromboembolic complications occurred. The time required for dissolution varies depending on the integrity of the filler and the state of the anatomical vascular bed. There were no significant differences between the arterial and venous, high-flow and low-flow, high-pressure and low-pressure, and high-resistance and low-resistance systems. Jean et al. proposed the hypothesis that solubility is positively correlated with the total amount of poloxamer and blood contact ratio [61]. According to this hypothesis, the better the vascular bed is filled, the longer the occlusion time.

### 3.2. Bone

Reconstruction of injured bone is currently a major challenge in the field of tissue engineering. If the bone does not heal completely, it may lead to permanent functional disabilities. GFs, mesenchymal stem cells, and bone-forming proteins are widely used in bone-repair applications [63,64,65,66,67,68]. However, significant cell death or loss of GFs can occur after the injection of these substances alone due to inflammation and a lack of matrix support. Therefore, to retain the proper number of cells and GF, a suitable delivery vehicle is needed for local use at the bone site. An ideal bone scaffold has the role of optimizing bioactive agents and cell delivery, diffusion of nutrients, oxygen, and metabolic waste. Poloxamer hydrogels are similar to ECM and exhibit high water content, high porosity, and structural flexibility [64]. It also has good hydrogel stability, prolongs the detention time of bone-regenerating agents at bone defect sites, provides local treatment, and precisely targets bone tissue repair. Thus, poloxamer can be used as an osteoconductive scaffold, allowing bone tissue to regenerate and grow at the site of bone tissue.

**Figure 6:**
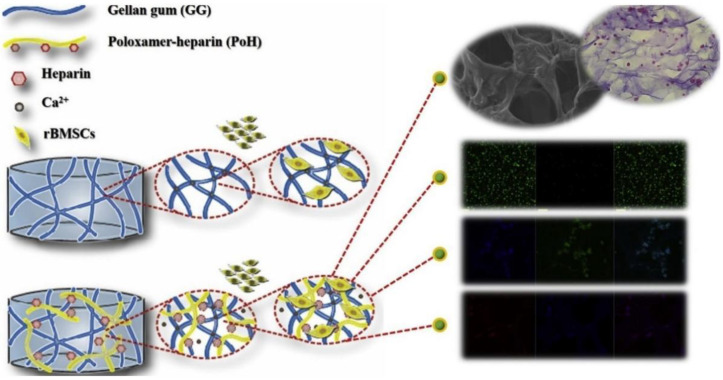
Double network hydrogel system of gellan gum hydrogel and poloxamer heparin hydrogel with rBMSCs encapsulation. The image has been taken from the publication by Choi et al. [64] with permission.

At a cellular level, poloxamers improve cellular affinity. The poloxamer hydrogel improved the hydrophilicity of the scaffold system and maintained osteoblast activity [65]. In addition, the rough surface and complex network of the hydrogel microenvironment induce high cell attachment and improve cell–scaffold interactions [64]. Liu et al. showed that poloxamer 188 promotes the adhesion and proliferation of mouse embryonic fibroblasts when used at concentrations of 5% wt or higher [69]. This result suggests that there is a minimal mixing ratio of poloxamer 188 that promotes cell attachment and proliferation. The internal structure of the hydrogel is porous and interconnected. The adhesion of cells to the surface decreases as pore size increases [70,71]. Joo et al. prepared a poloxamer-heparin/gellan gum double-network hydrogel (PoH/GG DNH), and the pore size of the gel scaffold decreased significantly with the increasing specific gravity of the poloxamer-heparin (Figure 6) [64].

In addition, poloxamer 188 improves the expression of osteogenic and chondrogenic genes, thereby promoting human dental embryonic stem cell differentiation [68]. However, some studies have indicated that poloxamers at the bone defect sites may negatively affect the initial healing procedure. Poloxamer 407 slow-release demineralized bone matrix (DBM) study showed that poloxamer inhibits the differentiation of mesenchymal stem cells but can promote cellular appreciation [72]. A study of 407-loaded lactoferrin (LF) with poloxamer also showed that poloxamer inhibited osteoblast differentiation early at the defect site and no increase in osteoblast viability was observed [65]. Mahboubeh et al. observed a similar increase in osteoblast viability and proliferation when rosuvastatin/chitosan/chondroitin sulfate nanoparticles were incorporated into a poloxamer 407 hydrogel [73].

Determined by the immune response of THP-1 human monocyte-like cells, the safety of poloxamer compared with suture materials was assayed by proinflammatory cytokine levels of LF/ poloxamers in vitro. During natural healing, the cytokine levels peaked within 24 h. After 48 h, the cytokine expression decreased in the presence of poloxamer, and the inflammatory response around the poloxamer was less severe than that around the surgical suture material [65].

At the tissue level, Kim et al. repaired the cuffs in a rat model using a poloxamer. Compared to a gelatin sponge, poloxamer exhibited a mature healing process of collagen fibers at an early stage and showed superior biomechanical properties at an early stage, which facilitated bone healing [74]. Despite the long degradation time, the residual poloxamer does not affect the bone healing process; over time, the residual poloxamer can be completely replaced by newly formed bone [66].

### 3.3. Cartilage

Joint cartilage deficiency is typically attributed to articular-related trauma and diseases. Currently, clinical cartilage restoration methods such as surgical interventions and autologous and allogeneic tissue grafts have several limitations in cartilage treatment. The efficacy of poloxamer-based tissue engineering solutions has been proven as well as traditional ones.

**Figure 7:**
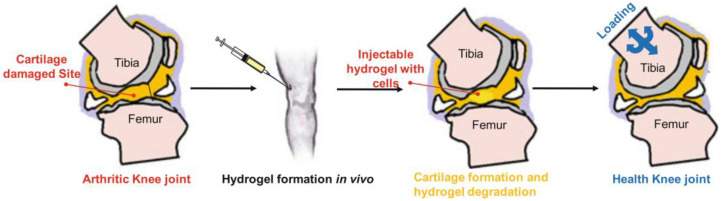
Schematic overview of cartilage tissue engineering using injectable hydrogels. The image has been taken from the publication by Dehghani et al. [32] with permission.

Hydrogels applied for cartilage regeneration must possess the ability to withstand the applied pressure (Figure 7). Poloxamer hydrogels are commonly used in combination with polymers or composite materials that contain mineral nanoparticles. Liu et al. implanted chondrocytes into an alginate-poloxamer/silk fibroin hydrogel and assayed cell viability to assess the applicability of this hydrogel [75]. Chondrocytes showed higher motility after seven days of culture, and once a stable pathway including a supply of nutrients and discharge of waste was created within the hydrogels, cells implanted inside the hydrogels multiplied comparatively faster. Thus, a favorable 3D environment could be created to support the growth of chondrocytes, preserve the chondrocyte phenotype, and promote their progression to cartilage-forming tissue.

Morille et al. constructed a poly (lactic-co-glycolic acid)/poloxamer 188/poly (lactic-co-glycolic acid) (PLGA-P188-PLGA)-based scaffold that releases trans-forming growth factor 3 (TGF-β3), which drives adipose tissue-derived mesenchymal stem cells (ASCs) to chondrocyte differentiation, as well as inducing increased expression of cartilage matrix-specific secretory factors [76]. Poloxamer 188 promotes cell proliferation. In addition to enhancing protein protection, p188 also plays an active role in cartilage formation. Poloxamer 188 protects against chondrocyte necrosis and stimulated chondrocyte matrix synthesis [76,77,78,79]. Cao et al. used a poloxamer as a scaffold combined with polyglycolic acid and calcium alginate to shape a naturally resilient cartilage-like tissue [80].

### 3.4. Nervous System

The nervous system comprises the central nervous system (CNS) and the peripheral nervous system (PNS). The CNS, consisting of the brain and spinal cord, is the control center of the physical body, while the PNS includes nerves and sensory organs. Neurological dysfunction may be caused by physical injury or neurodegenerative diseases [81]. Following nerve injury, activated astrocytes become reactive astroglia [61]. Reactive astrocytes increase the expression of several proteins that inhibit axonal growth in the vicinity of the injury. These changes modify the microenvironment in and around the injuries, inhibit neuronal cell growth and axonal regeneration, and affect the repair and regeneration of neural tissue. The capacity of the CNS to self-regenerate following injury is restricted owing to the poor ability to replace injured neurons and a highly inflammatory response around the site of injury. Reactive astrocytes are the major barrier to neuronal axonal regeneration. Comparatively, the peripheral ganglia show a superior ability for axonal regeneration after injury. However, in cases of chronic traumatic injury, tubes are needed to fill the gap and provide a suitable microenvironment within which axons can regenerate [81].

For central nerve injuries, poloxamers have some initial stability as a biomaterial, and after spinal cord injury, they can provide physical support to the injured spinal cord. Poloxamer 188 was used to treat ischemic spinal cord injuries [82]. Its effects are based on the mechanisms associated with hydrophobicity, tackiness, and rub resistance. By attaching to damaged cells and biomolecules such as fibrin, poloxamer 188 can hinder adhesion and friction between blood cells and blood vessel walls and prevent thrombosis [83]. Modification of the poloxamer by heparin enhances the affinity of the scaffold for NGF. The application of NGF to the site of spinal cord injury has been proven to be beneficial for the regeneration of axons. Studies have shown that the use of NGF-heparin-poloxamer hydrogels promotes axonal regeneration and the production of new capillaries, thus facilitating the treatment of spinal cord injury repair [84].

**Figure 8:**
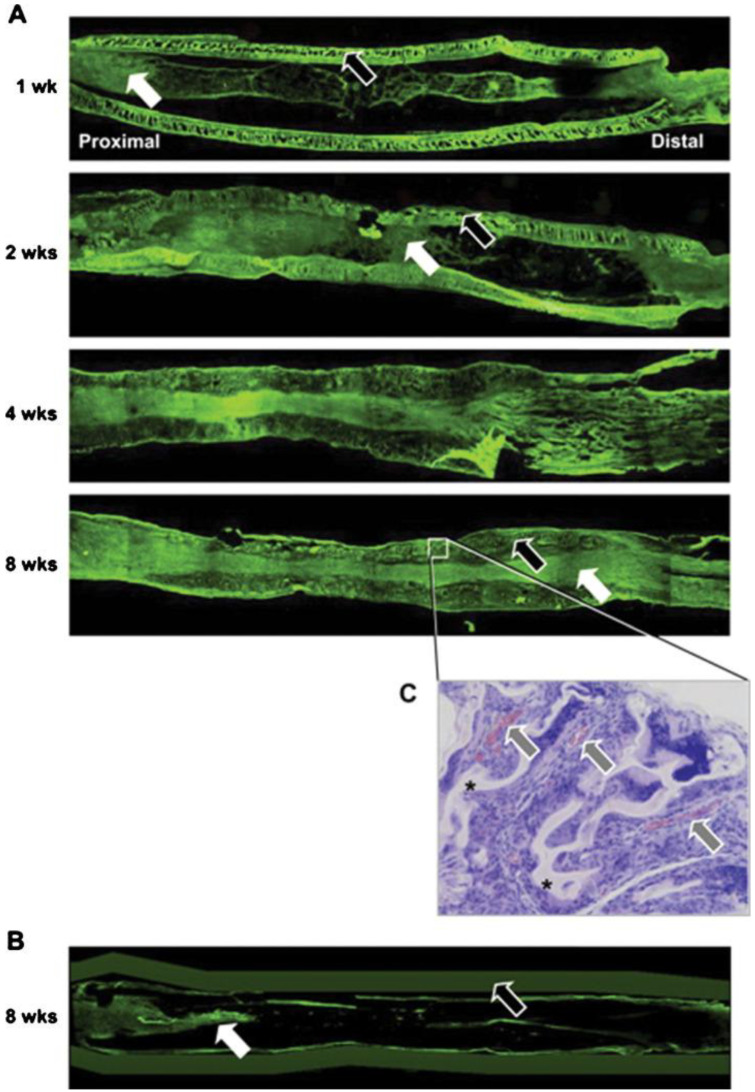
Nerve regeneration using (**A**) PLGA/F127 and (**B**) silicone tubes (white arrow shows regenerated nerve; black arrow shows tube wall) and (**C**) cross-sectional perspective of PLGA/F127 tube wall exhibiting the presence of blood vessels infiltrated within the wall. Gray arrow denotes blood vessel; * denotes PLGA/F127. The image has been taken from the publication by Kim et al. [22] with permission.

Polymer tubes have attracted considerable attention in the case of peripheral nerve injuries because of the disadvantages of both homografts and autografts. This semi-permeable nanoporous structure of the PLGA/Pluronic F127 nerve catheter inhibits the infiltration of fibrous tissue and allows for the exchange of nutritional substances and waste products. In addition, microscopic pores (approximately 50 μm in diameter) cover the outer surface, allow blood vessels to grow inward, and provide nutrition for axons growing within the guide tube (Figure 8). In addition, this scaffold was coupled with a low-intensity pulsed ultrasound. Physical stimulation is more conducive to nerve fiber growth. The asymmetric porous structure has selective infiltration, hydrophilicity, and ductal structure stability, which, combined with mechanical irritation, creates a suitable microenvironment within which neural repair can occur. PLGA/Pluronic F127 nerve conduits allow nerve fiber growth and exchange of nutrients with metabolic wastes. Curry et al. reduced neuronal damage in a rat model using poloxamer [7]. Poloxamer had a protective effect on excitability within the striatum of rats. Poloxamer shows neuroprotective effects through surface-active substances embedded in neuronal cell membranes, while it decreases the death of excitatory cells by disturbing the rupture of necrotic membranes and decreasing cell death to a low level in apoptotic cell death. Heparin-modified hydrogels can deliver multiple GFs locally and in a controlled manner through self-assembly. The hydrogel combined with GFs, such as fibroblast growth factor (bFGF) and NGF (15µg each), promoted axonal and myelin recovery, microtubule stabilization, and the proliferation of Schwann cells after sciatic nerve injury in diabetic rats [85].

### 3.5. Skin

The skin is composed of a keratinized layered epidermis and collagen-rich dermal connective tissue, which is the largest organ of the human body. The ECM interwoven with collagen fibers is the main component of the dermis. Effective treatment of skin wounds is an important aspect of tissue engineering, especially in the case of burns and wounds. Skin wound healing is a complicated process: 1. hemostasis and coagulation, 2. neovascularization and fibroblast differentiation; 3. granulation tissue formation; 4. tissue remodeling. Therefore, the ideal environment for rapid healing must meet the requirements for wound wetting and prevention of cell dehydration [86].

At present, the most common method is to manufacture 3D scaffolds based on the shape of tissues [86,87]. In the case of skin damage, the scaffold should satisfy the requirements of elasticity, moisture, pH, antibacterial performance, and wound healing. Hydrogels are cross-linked network structures that expand when they contain large amounts of water. Poloxamer hydrogels have important biomedical applications as scaffold poloxamers can promote wound healing because of their inherent pharmacological properties and biocompatibility [88]. It also exhibits degradability and high porosity. The simple use of drugs and therapeutic cell growth factors can promote wound healing, but it is easily degraded by proteases in the inflammatory microenvironment. However, poloxamer hydrogels provide an environment for the slow release of drugs or growth factors on the wound surface, preventing protease hydrolysis [89].

The poloxamer hydrogel fills the subcutaneous defect area, allowing the surrounding primary cells to grow while maintaining a large amount of tissue fluid, promoting the diffusion of nutrients and cell wastes through the scaffold, effectively healing wounds quickly and reducing scar formation. Pan et al. prepared poly (e-caprolactone-co-lactide)/poloxamer (PLCL/poloxamer) nanofibers. PLCL/poloxamer nanofibers showed better biocompatibility than PLCL scaffolds alone. This is mainly because of the introduction of poloxamers to construct hydrophilic nanofibers, which are interconnected and have a solid-phase structure that supports cell migration and proliferation. The PLCL/poloxamer nanofiber layer can be linked to the host skin tissue to become a protective barrier to the outside world, simulating the physical structure of normal skin [90]. Poloxamer/chitosan/hyaluronic (P407/CTS/HA) hydrogel shows epidermis, dermis, and stratum corneum similar to skin in wound healing of skin burns [88]. Studies have found that poloxamer hydrogels can reduce the secretion of inflammatory factors in skin wounds and play a role in inhibiting inflammation and repairing injuries [91].

Because of the plasticity and adhesion of poloxamer, compared with oral drugs, the half-life of poloxamer gel preparations containing drugs by subcutaneous injection is longer, which can extend the time medications stay on the skin’s surface [92]. They can be attached to local wounds without loss. Therefore, the combined application of poloxamer and growth factors or drugs can significantly promote skin tissue regeneration.

### 3.6. Organ

#### 3.6.1. Uterus

Intrauterine adhesion (IUA) refers to the replacement of the endometrial stroma with fibrous tissue when injured by trauma or surgery. Infertility, recurring miscarriages, abdominal pain, and other negative pregnancy outcomes are possible causes of severe intrauterine adhesions [93]. At present, the clinical treatment of IUA is mainly surgical resection of connective tissue and physical separation. Clinical evidence suggests that the prognosis of IUA is poor, which may be due to the failure of endometrial regeneration, resulting in a recurrence rate as high as 62.5%.

**Figure 9:**
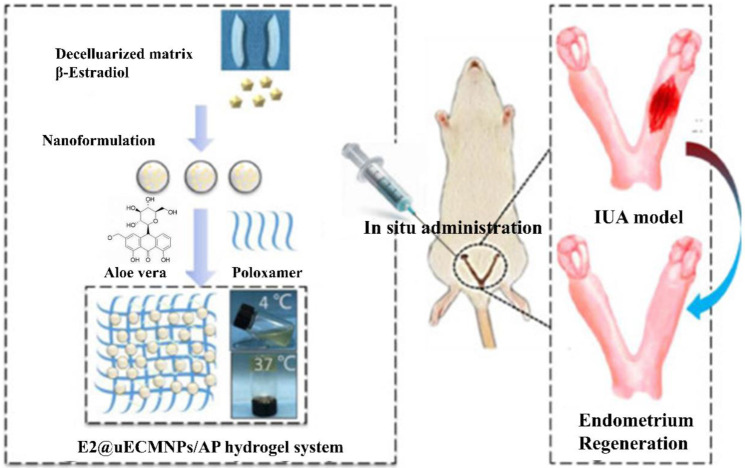
Schematic graphs of E2@uECMNPs/AP hydrogel system to promote endometrial regeneration for the prevention of IUA. The image has been taken from the publication by Yao et al. [93] with permission.

The poloxamer hydrogel is liquid at low temperatures, which is below 20 °C and has favorable injectability. The hydrogels will solidify when put into the uterine cavity or heated to 37 °C. Through the curing process, a hydrogel system can improve nanoparticle dispersion stability and greatly reduce agglomeration. Furthermore, several investigations have shown that poloxamer hydrogels exhibit good stability. In the early stages of damage, the hydrogel acted as a physical barrier. Decellularized uterus-derived nanoparticles (UECMNPs), β-estradiol (E2), aloe-poloxamer (AP), and other elements can function together to promote healing and endometrial regeneration (Figure 9) [93]. The hydrogels can not only supply E2 to the injured location but also have a synergistic effect on wound healing and repair of the damaged uterus’s morphology, structure, and function. Hydrogels have the ability to boost the concentrations of drugs in the target region while also extending the action time. Under the action of the poloxamer hydrogel, damaged cells can promote cell proliferation and self-repair, and cell activity is obviously enhanced.

Poloxamer hydrogel has dual functions as a carrier, increases cell sensitivity, and is expected to be a candidate drug for drug delivery. However, drug-derived nanoparticles appear to readily agglomerate under aqueous conditions after 24 h, which could impact drug release after storage.

#### 3.6.2. Lung

Protecting, repairing, and regenerating damaged lung tissues require innovative methodological strategies. Mechanical ventilation (MV) is the most prevalent approach used in clinical practice for the treatment of acute respiratory distress syndrome (ARDS). It allows for adequate gas exchange, while the respiratory muscles can stop working [94]. However, MV support therapy can cause pulmonary edema, which in turn can lead to lung damage [95]. The question of reducing ventilator-induced lung injury (VILI) has been discussed for several years. To solve this problem, Maria et al. explored the effect of poloxamer 188 on increasing the healing of alveolar resident cells in ventilator-injured lungs [96]. Damaged cells were quantified in the model by performing injurious or protective ventilation on the lungs of live and isolated rats, using a method of membrane-impermeable product improvement, and an observational analysis of alveolar cells in monolayers damaged by external forces was performed. Poloxamer 188 enhances the repair of the plasma membrane of alveolar-resident cells and shields the cell membrane from external injuries. However, the ex vivo and in vivo results were completely different. As a result, more research on the use of poloxamer to protect and repair damaged lung cells is required.

#### 3.6.3. Brain

Traumatic brain injury (TBI) is a primary cause of death and morbidity among children and adolescents. Every year, more than 1.7 million people in the United States suffer from traumatic brain injury. A small number of people continue to experience cognitive deficits, memory loss, and motor impairment over time, and the persistence of these symptoms has a significant negative impact on the health and psychological well-being of the injured person [97]. TBI is also associated with primary mechanical and secondary injuries. Secondary injury is the death of nerve cells due to a series of changes such as neuroinflammation and apoptosis, followed by primary mechanical injury. One can take advantage of the time before a secondary injury occurs to treat the patient in advance and to reduce or avoid the negative consequences of a secondary injury.

To date, only conventional medical interventions and care are available, and there are no effective treatments or medications for traumatic brain injury dysfunction. Purified poloxamer 188 has potent cellular anti-inflammatory and protective actions in pharmaceutical and clinical fields. Therefore, a study was carried out to determine how purified poloxamer 188 affects the recovery of sensory, motor, and cognitive skills in rats with controlled cortical-impact-induced TBI. Sensory and cognitive functions of adult rats were assessed by intravenous infusion of purified poloxamer 188. The results of these studies showed that purified poloxamer 188, when administered to rats 2 h after injury, reduced the size of damaged tissue, protected it, and improved sensory and cognitive functions in rats [98]. This means that purified poloxamer 188 can treat the dysfunction of post-traumatic brain injury and can protect and repair damaged brain cells and neural tissues. In addition, TBI-induced neuronal damage leads to incomplete cell membranes and impaired plasma membrane permeability. Studies have found that pharmacological interventions such as poloxamer 188 can reduce mitochondrial and lysosomal membrane damage by inhibiting apoptosis and restoring the original tight junctions and function of cells to seal damaged cell membranes [99], reduce neuronal death, and reduce damage to the blood-brain barrier caused by TBI, thereby reducing cerebral edema [82,100]. Further studies have revealed that poloxamer 188 activates autophagy during plasma membrane resealing after TBI; however, this conclusion still needs to be further investigated [101].

In general, the application of poloxamer in the field of brain repair and regeneration is mainly through the sealing of cell membranes, restoration of blood-brain barrier integrity, anti-inflammatory action to inhibit cell death, protection of damaged cells and nerves, and treatment of traumatic brain injury. The next few years hold great promise for research on the optimal dose and most appropriate timing for the use of poloxamer.

### 3.7. Others

#### 3.7.1. Adipose Tissue

Each year, millions of cosmetic procedures are performed to reconstruct soft tissue damage caused by injuries, tumor resection, deep burns, hereditary and congenital defects, and breast repair after tumor resection. Therefore, in current research, the promotion of adipose tissue engineering has become a hotspot. Because adipose tissue-derived mesenchymal stem cells (ASCs) qualify the conditions to differentiate into adipocytes, subcutaneous tissue can isolate them, expand in cell culture, and have little immune rejection by the organism. It is necessary to exploit biomaterials with favorable injectability or 3D-printed scaffolds to load adipose mesenchymal stem cells to repair soft-tissue defects [102].

Single-component poloxamers can provide the ideal microenvironment required for adipose tissue engineering. A barrier limiting its use is its low bioactivity; therefore, it needs to be blended with biocompatible polymers, such as oligopeptides, to form composite scaffolds for in situ regeneration of adipose tissue. In a comparative study, a single-component poloxamer hydrogel loaded with ASCs was studied in a hybridized hydrogel with a self-assembled oligopeptide containing an amino-acid sequence. Although physical cross-linking occurred in mice injected with a poloxamer hydrogel, this strength was not sufficient to resist dissipation in mouse endosomes, resulting in a late adipose regeneration time. In addition, the aggregation of cells in the hydrogel can also lead to the failure of adipose regeneration. However, adipose regeneration was observed in the composite hydrogels, probably due to the increased viscosity of the composite hydrogels. Therefore, it delays the dissipation of the hydrogels in mice, providing enough time for the differentiation of ASCs and the formation of adipose tissue. In addition, vascularization is important in adipose tissue. Hydrogels doped with poloxamers facilitated the repair of capillaries [103].

#### 3.7.2. Ligament

The anterior cruciate ligament shows poor healing. The critical mechanisms for its failure to heal are due to various factors, including hypocellularity, hypervascularity, and the lack of blood clots at injury sites to anchor the ligament ends and aid in tissue growth. Therefore, these soft tissues require surgical repair after injury [104].

Degradable ligament tissue engineering scaffolds need to accurately control their mechanical and degradation properties and modify their own to match their unique ligaments. Thus, scaffolds must mimic the behavior of ligaments to effectively replace ligaments.

The scaffold made of poloxamer microfibers wound and braided resembled the mechanical properties of the human anterior cruciate ligament. They are able to withstand cyclic mechanical loads as well and have high biocompatibility with C3 mesenchymal stem cells (MSCs). When cells were cultured in an induction medium at the appropriate cell seeding density, poloxamer scaffolds further supported the differentiation of MSCs into the ligament fibroblast phenotype. Eventually, periodic mechanical stimulation of the scaffold further stimulates MSC differentiation. The increased expression of ligament cell line markers, especially sclerosing axes, a vital marker of ligament and tendon differentiation, demonstrates the potential of this scaffold to bind mechanical stimulation for future progress in ligament tissue engineering [104].

#### 3.7.3. Skeletal Muscle

Owing to the large voltage gradients that can exist in these long cells, nerves and muscles are particularly sensitive to electrical shocks. Victims who are shocked experience nerve and deep muscle damage, mild signs of heat damage to the contact area, and in electroporated tissue, perforated cell membranes that can affect normal metabolic functions. If membrane damage can be repaired, cells can regain function and rebirth.

Poloxamer 188 has been shown to reduce the membrane damage caused by electroporation and improve tissue survival. Poloxamer 188 also reduces and promotes tissue recovery after chemical, mechanical, radiation, thermal damage, and ischemia-reperfusion injury to a variety of tissue types, such as neuronal, muscle, and cardiac tissue [105]. Table 2 provides an overview of all the studies we included in the application of poloxamers in tissue engineering.

## 4. Conclusions and Future Perspective

Tissue engineering is an emerging discipline for performing in vitro or in vivo construction of tissues or organs with a focus on coupling cells and scaffolds. The efficacy of poloxamers as multifunctional bioactive scaffolds in tissue engineering and bio-inks in 3D printing has been widely demonstrated and verified. Poloxamer is a water-soluble nonionic triblock copolymer with low toxicity, biodegradability, and biocompatibility, characterized by reversible thermal responsiveness and micellar self-assembly behavior. Poloxamer hydrogel is similar to ECM with high water content, high porosity, injectability, and structural flexibility.

In this review, we focused on poloxamer’s positive roles used as injectable hydrogel alone and poloxamer-based composite scaffolds combined with other biomaterials in vascular regeneration, wireless suture, artificial and temporary obstruction, cartilage, bone, skin, the nervous system, uterus, lungs, and brain. We concluded that poloxamers can assemble their own structures by binding a range of substances such as cells, GFs, and drugs, restricting the diffusion of substances, maintaining their integrity, improving cell viability, and transporting them to the designated site through the properties of the hydrogel itself. It allows the scaffold to act as a cell carrier and a drug micro-nanocarrier to regenerate the damaged tissue. Moreover, the scaffolds will create a suitable microenvironment for cell adhesion, proliferation, and differentiation. Moreover, they can also inhibit platelet activation and reduce the friction between blood cells and the blood vessel wall owing to the viscidity. When poloxamer hydrogels cannot meet the repair requirements, they are usually mixed with additives to create composite scaffolds. Commonly used additives include bioactive glasses, HA, silk fibroin, PLGA, heparin, etc.

Poloxamer can provide high bioactive surface structures and sites and significantly improve the hydrophilicity of the scaffold system.

Although these aspects have been used and great achievements have been made, it still needs to be further studied and enhanced in clinical treatment. The proposed future development directions are as follows: explore mixing or blending with more types of polymers or inorganic filler materials which can better meet the requirements for biomaterials in the future due to their more advantages and adjustable properties; develop more high-performance tissue engineering scaffold materials compatible with poloxamer, which can achieve better biocompatibility, biodegradability, and mechanical properties, and exquisitely imitate the complex composition, structure, and function of tissues; exploit more effective structural design that can adapt to the cell microenvironment to promote tissue repair; improve the 3D-printing accuracy and meet the biomimetic nanoscale so as to achieve more effective repair of tissue structure and function. It is also hoped that the exploration of the comprehensive application of various new technologies will contribute to the fabrication of novel poloxamer-based scaffolds with interesting properties.

## Figures and Tables

**Table 1:** The properties of the common [PEOx- PPOy- PEOx] copolymers. The table has been taken from the publication by Zarrintaj and Russo et al. [7,11] with permission.

Poloxamer	Pluronic	PEO%	Viscosity (Pa·s)	Surface Tension (Dyn cm^−1^)	Average Molecular Weight	Melting Point (°C)	* HLB	CMC	Cloud Point	Application
P105	L35	50	0.375	49	1900	7	18–23	5.3	73	Surfactant, used for synthesizing the copolymer
P123	L43	30	0.310	47	1850	−1	7–12	2.2	42	Surfactant, drug encapsulation
P124	L44	40	0.440	45	2200	16	12–18	3.6	65	Surfactant, cosmetics, and pharmaceuticals applications
P182	L62	20	0.450	43	2500	−4	1–7	4	32	Nonionic surfactant, delivery system
P184	L64	40	0.850	43	2900	16	12–18	4.8	58	Surfactant
P188	F68	80	1.000	50	8400	52	>24	4.8	>100	Hemorheological activity, antithrombotic, cell membrane closure, phagocytic activation (stimulating phagocytosis and superoxide anion production), and neutrophil threshing. Increased expression of osteogenic and cartilage genes
P234	P84	40	0.280	42	4200	34	12–18	7.1	74	
P235	P85	50	0.310	42	4600	34	12–18	6.5	85	Inhibition of multidrug resistance
P237	F87	70	0.700	44	7700	49	>24	9.1	>100	Scaffold, delivery system
P238	F88	80	2.300	48	11,400	54	>24	2.5	>100	Regulation of erythrocyte aggregation
P288	F98	80	2.700	43	13,000	58	>24	7.7	>100	Regulation of erythrocyte aggregation
P333	P103	30	0.285	34	4950	30	7–12	6.1	86	Body and hand creams, lotions
P334	P104	40	0.390	33	5900	32	12–18	3.4	81	Hair tonics, dressings, delivery system
P335	P105	50	0.750	39	6500	35	12–18	6.2	91	Delivery system, breath freshener, and mouthwash
P338	F108	80	2.800	41	14,600	57	>24	2.2	>100	Surfactant, coating
P403	P123	30	0.350	34	5750	31	7–12	4.4	90	Inhibition of multidrug resistance, administration
P407	F127	70	3.100	41	12,600	56	18–23	2.8	>100	Stimulation of macrophages, controlled-release gels, stimulation of EGFc production, tissue engineering, long circulation particles

* HLB: hydrophilic–lipophilic balance.

**Table 2:** Poloxamers in tissue engineering applications.

References	Scaffold Materials	Additive	Site of Action	Types	Main Function	Shortcoming
[106]	Simvastatin Poloxamer 407 Hydrogel	Ti-6Al-4V Scaffolds	Bone vascularization	Thermosensitive hydrogel	Improve neovascularization, osseointegration, and bone ingrowth	The injected thermosensitive biodegradable hydrogel blocked the pores
[55]	Poloxamer 407 BSA	Heparin	Arterial vascular stumps	Gel	Heparinized P407 can be applied as an endovascular scaffold for tissue adhesive anastomosis without suture	Residual heparin is probably at the anastomosis
[61]	Poloxamer 407	contrast agent and food coloring	Arteries	Gel	An adjunct tool for devascularization	The effect of injections affects the gel effect
[60]	PU/PCL/Poloxamer	DMF and THF	Small diameter PU/PCL The electrostatic spinning hollow tube	Double hollow tube with hydrophilic inner PU/PCL /Poloxamer	Promote cell adhesion and cell proliferation and inhibit platelet adhesion in vascular engineering.	
[56]	Poloxamer 407	Cyanoacrylate	Venous vessel stumps	Gel	Keep the venous cavity open and prevent it from collapsing. Promise precise access to the vessel stump and lower the risk of glue penetration into the lumen	
[54]	PLGA: poloxamer 188	FGF-2 or PDGF-BB	Biological fluids	Nanoparticle system	PLGA: poloxamer nanoparticles are stable, non-toxic, and can be effectively freeze-dried for long-term storage in simulated biological fluids. The nanosystem preserves the bioactivity of the encapsulated GFs	
[63]	Poloxamer 407/collagen sponge	rhBMP-2	Rat Mandible	Hydrogels	Maintains the protein in situ and has a chemotactic effect on mesenchymal cell differentiation.	Residual, but does not affect the bone healing process
[64]	Poloxamer-heparin/gellan gum double network hydrogel	rBMSCs	Under the dorsal subcutaneous region of the nude rat.	Hydrogels	A moderate increase in poloxamer enhances cell adhesion and proliferation	
[107]	PCL/gelatin/Poloxamer 188			Electrospinning scaffold	Ability to enhance osteogenic performance	
[65]	Poloxamer 188/Poloxamer 407	Lactoferrin	Rat skull	Hydrogels	Maintain the viability of osteoblasts	Early inhibition of osteoblast differentiation
[108]	Poloxamer 407	Holmium-Containing Bioactive Glasses		Hydrogels	Glass ions facilitate the micellization of poloxamers. Promotes preosteoblast proliferation and osteosarcoma cell death	
[74]	Poloxamer 407		Rat shoulder	Hydrogels	P407 promotes the number and maturation of collagen fibers	
[106]	Titanium alloy	Simvastatin- Poloxamer 407	Rabbit tibia	Scaffold	Promotes new bone expansion and neovascularization	
[66]	Poloxamer 407	BMP Excipients	Rat hind leg	Hydrogels	Poloxamer delivers BMP better than other carriers	
[109]	Poloxamer 407	BMP	Rabbit Femur	Hydrogels	Promote osteoblast differentiation, inhibit osteoclast activity, and prevent continuous destruction of bone around the interface	
[72]	Poloxamer 407	Calcium Phosphate Ceramics	Rabbit skull		The incorporation of p407 does not hinder the bone repair ability and bone conductivity of cap ceramics	
[110]	HA/Poloxamer			Hydrogels	Increased Ha content enhances intermolecular chelation with calcium ions, promoting calcium phosphate nucleation and increased growth	
[68]	Pluronic P85/Pluronic F127/Pluronic F68	Human tooth germ stem cells		Solution	PF68 increases the pluripotency of stem cells to transform into osteogenic, cartilage, and adipogenic tissues	
[73]	Poloxamer 407/HA	Rosuvastatin-loaded chitosan/chondroitin sulfate		Hydrogels	Improve osteoblast viability and proliferation ability	
[67]	Bioactive Glass-Incorporated Alginate-Poloxamer/Silk Fibroin Hydrogels	IGF-1		Hydrogels	Maintain the biological activity of IGF-1	
[75]	Alginate-poloxamer/silk fibroin	Chondrocytes		Hydrogels	Maintenance of chondrocyte growth and preservation of chondrocyte phenotype	
[111]	Poloxamer/HA	SFN Sulforaphane		Hydrogels	Promotes cartilage protection in vitro and reduces osteoarticular inflammation.	
[76]	PLGA-P188-PLGA	TGF-β3		Hydrogels	Sustained protein release to improve hMSC survival	
[112]	Pluronic F68/F127	rAAV		polymer micelles	Gene repair of hMSCs to promote cartilage formation	
[113]	Poloxamer/HA	KGF-2	Rat left knee joint	Hydrogels	Improve articular cartilage morphology and inflammation, reduce proteoglycan loss	
[114]	Poloxamer 188/Poloxamer 407	GlcN		Hydrogels	GlcN released from gel binds to chondrocytes preferentially compared to the aqueous solution, reducing drug loss	
[115]	Poloxamer/hyaluronic acid	β-lapachone			Reduce secretion of pro-inflammatory molecule CXCL8 and restore synovial fluid rheological properties	
[85]	poloxamer 407 Heparin	bFGF/NGF	Axons and myelin sheaths of the sciatic nervous system	Gel	Own good affinity for large amounts of GFs and stably control its release and prevents in vitro degradation	
[84]	Hyaluronic acid/Poloxamer	NGF	Rat spine	Hydrogels	Reduce reactive astrocytes, inhibit axon regeneration protein, inhibit glial scarring	Functional improvement and regeneration of composite hydrogels did not reach control levels
[17]	Hyaluronic acid/Poloxamer	bFGF, NGF	Rat spine	Hydrogels	Improve neuronal survival, axonal regeneration, inhibition of reactive astrocytes, and repair of motor function in injured spinal cord	
[116]	PLGA-poloxamer 188-PLGA triblock polymer	SCAP and BDNF	Rostrally and caudally of rat spinal cord	Microspheres	Reduce inflammation.	
[88]	poloxamer 407/Chitosan/Hyaluronic acid	Vitamins A, D, and E	Flexor side of the left forearm of a human	Hydrogels	A weak acidic environment promotes fibroblast growth	
[89]	Poloxamer 407	FGF-21	Rat back	Hydrogels	Promote epithelialization and granulation tissue formation, own a good anti-inflammatory effect and promote cell value-added, accelerate the healing of burned skin	
[91]	Poloxamer/Chitosan/	ZnG/rhEGF@Chit/Polo	Rat back	Hydrogels	Reduce the secretion of the inflammatory factor IL-6, which has an anti-inflammatory effect	
[90]	PLCL/ Poloxamer Nanofibers and Dextran/Gelatin Hydrogels			Nanofibers	Good mechanical properties and support cell survival	
[117]	Poloxamer/Hyaluronic acid			Hydrogels	Enhance the accumulation of protein values in the wound area, increase permeability, and promote wound healing.	
[118]	Alginate/poloxamer			Hydrogels	Induce proliferation of human keratin-forming cells. Reduces local infection of wound inflammation and promotes healing	
[119]	Poloxamer 407and/or βCD-derivatives intended	Thpp		Foam	The presence of P407and/or βCD-derivatives promoted the diffusion of THPP in the foam.	
[92]	Poloxamer	2% doxycycline, 1% chloramphenicol, 0.5% mupirocin	Parrot wings	Hydrogels	Expand half-life and prolong retention of GFs on the surface of the skin through subcutaneous injection of the drug-containing poloxamer gel formulation compared to oral administration	
[103]	Poloxamer 407/octapeptide	ASCs	Rat neck	Hydrogels	Allow sufficient time for adipose tissue differentiation and promote capillary repair	The use of poloxamer gel alone had no positive effect on adipose tissue regeneration.
[104]	Poly(lactide)/poloxamer		Anterior cruciate ligament	Microfibers and scaffolds composed of twisted/ braided fibers		
[105]	Poloxamer 188		Electroporated muscle	Injection solution	Significantly reduce residual 99mTc PYP in electroporated skeletal muscle, reduce damage, and improve survivability	
[120]	Poloxamer 188 and Poloxamer 407	AgNPs	Root canal	Thermosensitive hydrogel	AgNPs-PL can remove the biofilm of enterobacter faecalis in dentin and dentin tubules.	

## Data Availability

In this paper, references are attached to all data, charts, and tables.
